# Mechanism of Penoxsulam’s Effect on Chlorophyll Synthesis and the Metabolism of Foxtail Millet

**DOI:** 10.3390/plants14081211

**Published:** 2025-04-15

**Authors:** Tingting Chen, Qi An, Ting Zhang, Siyu Yan, Jiaxing Li, Xie Song, Juan Zhao, Xiaorui Li, Chunyan Hu, Shuqi Dong

**Affiliations:** 1 College of Agronomy, Special Orphan Crops Research Center of the Loess Plateau, MARA, Shanxi Agricultural University, Taigu 030800, China; sxndctt@163.com (T.C.); a7n7q7i@163.com (Q.A.); zt1138514174@163.com (T.Z.); yan18235856798@163.com (S.Y.); lijiaxing147972@163.com (J.L.); sxndsxe@163.com (X.S.); sxndzhaojuan@163.com (J.Z.); lixiaorui@sxau.edu.cn (X.L.); 2 College of Plant Protection, Shanxi Agricultural University, Jinzhong 030800, China

**Keywords:** penoxsulam, foxtail millet, chlorophyll synthesis, chlorophyll metabolism, herbicide, phytotoxicity

## Abstract

Foxtail millet is a characteristic miscellaneous grain crop with many benefits in current agricultural production and is crucial in the adjustment of the planting structure and the sustainable development of dry farming. However, the harmful effects of weeds have become a critical challenge, restricting the modern production of foxtail millet. The effect of penoxsulam on the chlorophyll metabolism pathway of foxtail millet and its physiological mechanism was studied. Spraying penoxsulam on foxtail millet leaves significantly reduced the content of chlorophyll synthesis precursors (5-aminolevulinic acid (ALA), Porphobilinogen (PBG), Protoporphyrin IX (ProtoIX), Mg-protoporphyrin IX (Mg-ProtoIX), and Protochlorophyllide (Pchlide)). Moreover, the activities of key synthetic enzymes (magnesium chelatase (MgCh) decreased compared to control, while the activities of degrading enzymes (pheophorbide a oxygenase (PAO) and pheophytinase activities (PPH) increased significantly. The study revealed the mechanism of penoxsulam inducing crop phytotoxicity by interfering with the dynamic balance of chlorophyll metabolism, which provided a theoretical basis for the scientific application of herbicides and the study of foxtail millet drug resistance.

## 1. Introduction

Foxtail millet is a miscellaneous grain crop with many benefits in agricultural production and is crucial in the adjustment of the planting structure and the sustainable development of dry farming. In recent years, the whole-genome sequencing of foxtail millet has been conducted. Owing to its small genome, short growth cycle, easy reproduction, self-pollination, and C_4_ photosynthesis [1,2,3,4], it has become a new model plant for studying gramineous crops and has been extensively studied domestically and abroad [5,6]. Chemical weeding in crop fields is a convenient, effective, and reliable thinning method that plays a crucial role in agricultural production. Using herbicides to control weeds; however, causes certain phytotoxicity to crops, affecting crop physiology, biochemistry, growth and productivity [7]. Zhang et al. [8] found that when herbicides are sprayed on weeds, the responses increase as the dose increases; however, the increase in the herbicide dose may exceed the applicable scope of crops, causing uneven spraying and phytotoxicity to crops.

Changes in the pigment content represent an index of toxic substance-induced phytotoxicity [9]. Chlorophyll a and b (Chl a and Chl b) participate in the primary photosynthetic reaction because they absorb light energy [10]. Carotenoids (Car) are classified as C_40_ terpenoids that participate in biological processes, such as photosynthesis, photoprotection, and plant development as well as antioxidant defense systems that neutralise reactive oxygen species (ROS) under stress conditions [11,12]. Therefore, understanding the mechanism underlying the change in photosynthetic pigments is crucial for improving photosynthetic efficiency and crop yield [13,14]. Decreased chlorophyll concentrations were observed in aquatic plants exposed to atrazine [15,16,17].

**Figure 1:**
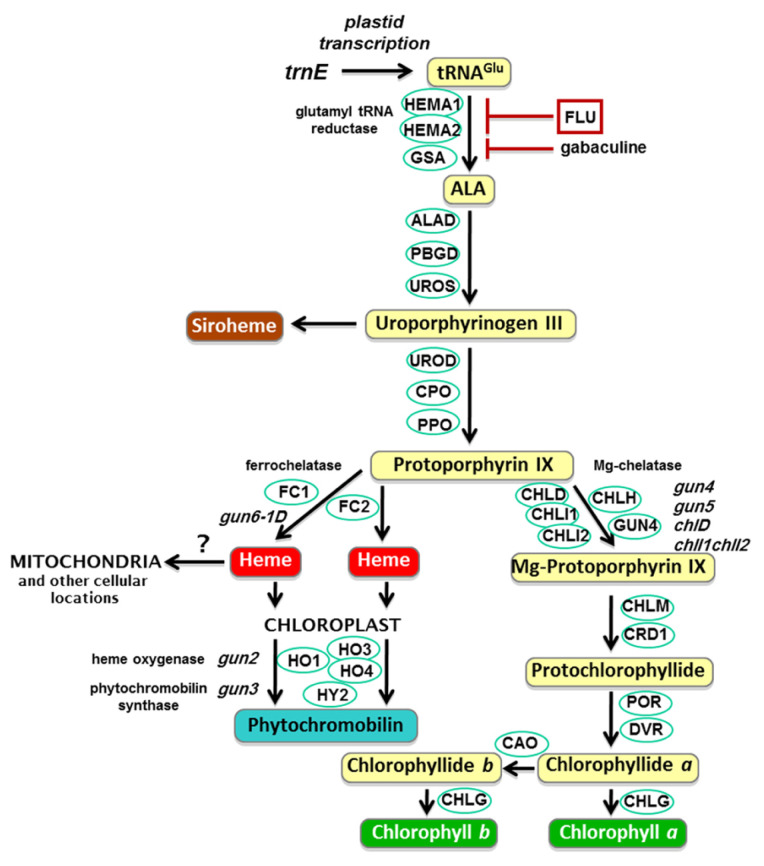
The plant tetrapyrrole synthesis pathway [25].

Chlorophyll biosynthesis in plants is a complex process involving many enzymes. The study of Chlamydomonas, Arabidopsis thaliana, and rice highlights that the entire chlorophyll biosynthesis process requires 15 reactions and 15 enzymes [18]. The biosynthetic pathway of chlorophyll is as follows: Glu > 5-aminolevulinic acid (ALA) > Porphobilinogen (PBG) > Uroporphyrinogen III (UroIII) > Protoporphyrin IX (ProtoIX) > Mg-protoporphyrin IX (Mg-ProtoIX) > Protochlorophyllide (Pchlide) > Chl a > Chl b (see Figure 1).

Magnesium chelatase (MgCh) is a key regulatory enzyme in the chlorophyll synthesis pathway. Similarly, magnesium is vital in chlorophyll synthesis; however, Chl a and Chl b are inseparable from magnesium [19]. The first enzyme to catalyse magnesium is the rate-limiting enzyme magnesium ion chelatase, which is crucial in chlorophyll synthesis [20]. This enzyme regulates the synthesis of Mg-ProtoIX [21]. MgCh is a multi-subunit complex enzyme, including CHL1 (40 kd), CHLD (70–80 kd) and CHLH (140 kd). In the CHLD enzyme, the most important auxiliary binding protein is GUN4 of MgCh [22,23,24]. Therefore, considering magnesium ion chelatase in the analysis of photosynthetic mechanism is essential.

**Figure 2:**
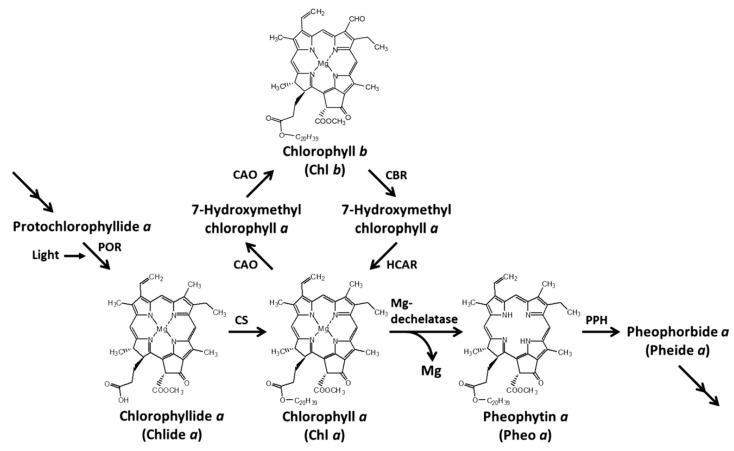
The plant tetrapyrrole synthesis pathway with key enzymes [35].

Leaf senescence is an adaptive mechanism for plants to a specific degradation process, which can be caused by external environmental stress or the internal mechanism adjustment of plants. This phenomenon is due to the fact that the amount of chlorophyll degradation in plants is higher than that of chlorophyll synthesis, thus weakening photosynthesis in leaves [26]. In Arabidopsis thaliana, the degradation of chlorophyll from a green product to a colourless intermediate pFCC requires the participation of many enzymes. At first, chlorophyll is dephosphorized by chlorophyllase and the metal-chelating substrate to become pheide a (Pheide a). Pheide a needs three enzymatic reactions: first, pheophorbide a oxygenase (PAO), then pheophytinase (PPH), and finally, pheide A is transformed by red chlorophyll metabolite reductase (RCCR), becoming a non-fluorescent chlorophyll metabolite stored in vacuoles [27,28,29] (see Figure 2). PAO catalyses the formation of chlorophyll decomposition products, green reduction, and colourless substance formation [30]. PPH also plays an important role in chlorophyll degradation. PPH is a chloroplast hydrolase that plays a regulatory role in senescence [31]. Studies by Jiang et al. showed that when rice leaves were in senescence, the expression of PAO gene was up-regulated, which promoted the decomposition of Chl, resulting in the decrease of Chl content in rice leaves [32]. Many studies have found that saline–alkali stress can destroy the chlorophyll metabolism of plants, inhibit the synthesis of ALA to Chl a, and cause symptoms such as leaf chlorosis, senescence and wilting. For example, saline–alkali stress significantly down-regulated the expression level of the porphyrinocholine deaminase gene PBGD in two rice varieties (saline–alkali-tolerant varieties and saline–alkali-sensitive varieties), up-regulated the expression of chlorophyllase gene, accelerated the degradation of Chl, and reduced the contents of Chl a, Chl b and total Chl [33]. Mitsuru’s research showed that salt stress inhibited CHl synthesis by up-regulating the expression level of the PPH gene in cucumber [34].

However, weeds are harmful to foxtail millet production and are sensitive to herbicides. To control gramineous weeds, the stems and leaves are sprayed with herbicides without legal registration. The herbicide penoxsulam has good control of gramineous weeds in paddy fields. In this study, penoxsulam was selected to control weeds in grain fields. The aim of the investigation is to study the effects of penoxsulam on chlorophyll anabolism in foxtail millet variety Jingu 21 and provide a theoretical basis for reasonable, safe, and efficient control of weeds in grain fields.

## 2. Results

### 2.1. Effects of Penoxsulam on the Precursors of Chlorophyll Synthesis in Foxtail Millet

The ALA content decreased on the foxtail millet variety Jingu 21 after being sprayed with increasing doses of penoxsulam (P0, P1, P2, and P3) at a 0, 15, 30 and 60 application rate/g a.i. ha^−1^), respectively.

**Figure 3:**
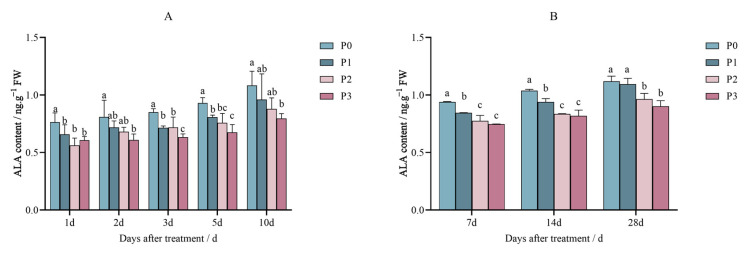
Effect of penoxsulam on ALA content of foxtail millet (**A**) pot experiment, (**B)** field experiment. Comparison between treatments of different concentrations on the same day, with lowercase letters representing a significant difference (*p* < 0.05). P0–P3 represent four different spraying doses of 0, 15, 30 and 60 g a.i. Ha^−1^, respectively.

Figure 3A shows that when Jingu 21 leaves were grown in a pot experiment and treated with increasing concentrations of penoxsulam, on the first day of the experiment, the ALA content decreased by 14.00%, 30.81%, and 20.54% compared to P0. After the second day of spraying penoxsulam on foxtail millet variety Jingu 21 leaves, we found that the ALA content decreased by 24.71% more with the P3 treatment than with the control P0. The ALA content in the P1 and P2 treatments was not significantly different from that in the P0 treatment. In further treatment with penoxsulam of Jingu 21 leaves, after the third day, the ALA content decreased by 16.02%, 18.40%, and 25.57% compared to P0. In the five-day experiment with penoxsulam sprayed on Jingu 21 leaves, the ALA content decreased by 13.20%, 21.15%, and 27.41%, respectively, more than P0. Finally. After the 10th day of spraying penoxsulam on Jingu 21 leaves with the P3 dosage, the ALA content decreased by 26.55% compared to control P0. The ALA content in the P1 and P2 treatments was not significantly different from that in the P0 treatment (Figure 3A).

Figure 3B shows the results of the experiment tested on field conditions when foxtail millet variety Jingu 21 leaves were sprayed with penoxsulam variety Jingu 21 leaves. Seven days after spraying penoxsulam, the ALA content decreased by 9.56%, 19.54%, and 21.15%, respectively, compared to P0. When the penoxsulam treatment was applied to Jingu 21 leaves, after the 14th day, the ALA content decreased by 9.56%, 19.54%, and 21.15%, respectively, compared to P0. On the 28th day after spraying penoxsulam on Jingu 21 leaves, the ALA content decreased by 13.81% and 19.31% under the P2 and P3 spraying doses, respectively, compared to P0 (Figure 3B).

The effect of spraying penoxsulam (P0, P1, P2, and P3) on Jingu 21 leaves grown in the pot experiment on the PBG content was that the decrease was gradual with an increase in the spraying dose.

**Figure 4:**
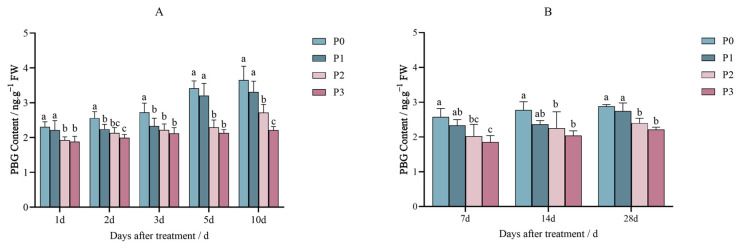
Effect of penoxsulam on PBG content of foxtail millet (**A**) pot experiment, (**B**) field experiment. Comparison between treatments of different concentrations on the same day, with lowercase letters representing a significant difference (*p* < 0.05). P0–P3 represent four different spraying doses of 0, 15, 30 and 60 g a.i. Ha^−1^, respectively.

Figure 4A indicated after the first day of spraying penoxsulam, the PBG content on Jingu 21 leaves decreased by 16.50% and 18.31% under P2 and P3 dosages, respectively, compared to P0. On the 2nd day after the treatment of penoxsulam sprayed on Jingu 21 leaves, the PBG content showed 12.32%, 16.67%, and 21.92% decreases compared to P0. Similarly, 3 days after treatment, the PBG content decreased by 14.31%, 18.40%, and 22.28% compared to P0. After the 5th day of treatment with penoxsulam sprayed on Jingu 21 leaves, the PBG content decreased by 32.88% and 37.50% under P2 and P3 dosages, respectively, compared to P0. At 10 days after treatment with penoxsulam on Jingu 21 leaves, the PBG content under the P3 dose decreased by 39.47% compared with P0; the PBG content in the P1 and P2 treatments was not significantly different from that in the P0 treatment (Figure 4A).

In the field experiment, after the treatment with penoxsulam on Jingu 21 leaves, the PBG contents of P1 dose and P0 showed no significant differences between 7 and 28 days after spraying penoxsulam. The PBG content decreased by 18.83% and 26.50% under the P2 and P3 sprayed doses, respectively, compared to P0 on the 14th day after treatment. At 28 days after treatment with penoxsulam on Jingu 21 leaves, the PBG content decreased by 16.85% and 23.27% under the P2 and P3 sprayed doses, respectively, compared to P0 (Figure 4B).

The ProtoIX content in Jingu 21 decreased after the penoxsulam (P0, P1, P2, and P3) treatment in the pot and field experiments, and the decrease trend was gradual with an increase in the spraying dose.

**Figure 5:**
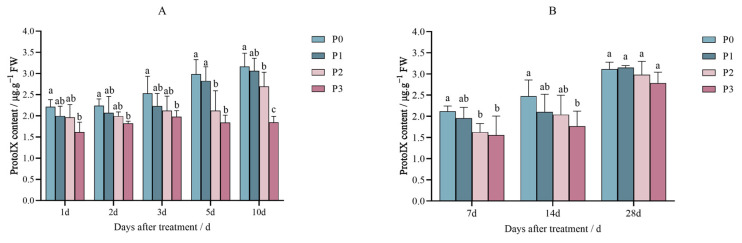
Effect of penoxsulam on ProtoIX content of foxtail millet (**A**) pot experiment, (**B**) field experiment. Comparison between treatments of different concentrations on the same day, with lowercase letters representing a significant difference (*p* < 0.05). P0–P3 represent four different spraying doses of 0, 15, 30 and 60 g a.i. Ha^−1^, respectively.

Figure 5A shows the results of the pot experiment of penoxsulam on Jingu 21 leaves; the ProtoIX content decreased by 26.94% under the P3 sprayed dose compared to P0 on the first day after spraying penoxsulam. The ProtoIX content in the P1 and P2 treatments was not significantly different from that in the P0 treatment. Two days after treatment with penoxsulam on Jingu 21 leaves, the ProtoIX content decreased by 18.63% under the P3 sprayed dosage compared to P0, while the ProtoIX content in the P1 and P2 treatments was not significantly different from that in the P0 treatment. In a further pot experiment, after 3 days of penoxsulam treatment on Jingu 21 leaves, there were no significant differences in the ProtoIX contents among the P1, P2, and P0 sprayed dosages. The content of ProtoIX under the P3 treatment decreased significantly by 21.84% compared with P0. After the 5th day of the pot experiment with penoxsulam dosages on Jingu 21 leaves, the ProtoIX content decreased by 28.83% and 38.24% in the P2 and P3 sprayed doses, respectively, compared to P0, while there was no significant difference in ProtoIX content between the P1 treatment and P0 treatment. Finally, after 10 days of treatment with penoxsulam dosages on Jingu 21 leaves, there were no significant differences in the ProtoIX content between P1 and P0. Compared with the P0 treatment, the content of ProtoIX in the P2 and P3 treatments decreased by 20.48% and 21.93% (Figure 5A).

Figure 5B shows the results of the field experiment, when penoxsulam dosages were sprayed on Jingu 21 leaves; after the 7th day, the ProtoIX content decreased by 23.24% and 26.52% under P2 and P3 dosages, respectively, compared with P0, while the ProtoIX content in the P1 treatment was not significantly different from that in the P0 treatment. In addition, after 14 days of treatment with penoxsulam on Jingu 21 leaves, the ProtoIX content was reduced by 28.55% under the P3 sprayed 167 dosages compared with P0, while the Proto IX content in the P1 and P2 treatments was not significantly different from that in the P0 treatment. Moreover, after 28 days of treatment with penoxsulam on Jingu 21 leaves, there were no significant differences in the Proto IX contents between P1, P2, P3 and P0 (Figure 5B).

The Mg-ProtoIX content in Jingu 21 decreased after treatment with penoxsulam (P0, P1, P2, and P3), and the decrease trend was gradual with an increase in the spraying dose.

**Figure 6:**
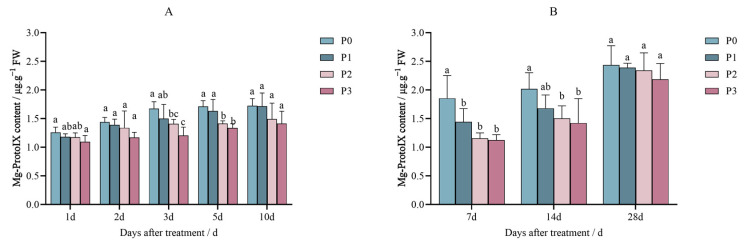
Effect of penoxsulam on Mg-ProtoIX content of foxtail millet (**A**) pot experiment, (**B**) field experiment. Comparison between treatments of different concentrations on the same day, with lowercase letters representing a significant difference (*p* < 0.05). P0–P3 represent four different spraying doses of 0, 15, 30 and 60 g a.i. Ha^−1^, respectively.

Figure 6A shows the results of the pot experiment; under different spraying dosages of penoxsulam on Jingu 21 leaves, the Mg-ProtoIX content showed no significant differences compared with P0 after 1 and 2 days of spraying penoxsulam. However, three days after treatment in the pot experiment with penoxsulam on Jingu 21 leaves, the Mg-ProtoIX content decreased by 15.85% and 28.02% under P2 and P3 sprayed doses, respectively, compared with P0; there was no significant difference between the P1 treatment and the P0 treatment. The Mg-ProtoIX content on the 5th day after treatment in the pot experiment with penoxsulam on Jingu 21 leaves decreased by 21.91% in the P3 treatment compared with P0. The Mg-ProtoIX content in the P1 and P2 treatments was not significantly different from that in the P0 treatment. On the 10th day after treatment in the pot experiment with penoxsulam on Jingu 21 leaves, the results show the contents of Mg-ProtoIX between P1, P2 and P3 were not significantly different from P0 (Figure 6A).

Figure 6B shows the field experiment. On the 7th day after spraying penoxsulam on Jingu, 21 leaves, the Mg-ProtoIX content under P1, P2, and P3 dosages decreased by 22.14%, 37.62%, and 39.36%, respectively, compared to P0. The field experiment continued after the 14th day of spraying penoxsulam on Jingu 21 leaves; the Mg-ProtoIX content decreased by 25.42% and 29.46% under P2 and P3 sprayed dosages, respectively, compared to P0. There was no significant difference between the P1 treatment and the P0 treatment. After 28 days of the field experiment of the treatment with penoxsulam on Jingu 21 leaves, Mg-ProtoIX contents showed no significant differences between all dosages tested (Figure 6B).

After the penoxsulam (P0, P1, P2, and P3) treatment, the Pchlide content in Jingu 21 decreased, and the decreasing trend was gradual with an increase in the spraying dose.

**Figure 7:**
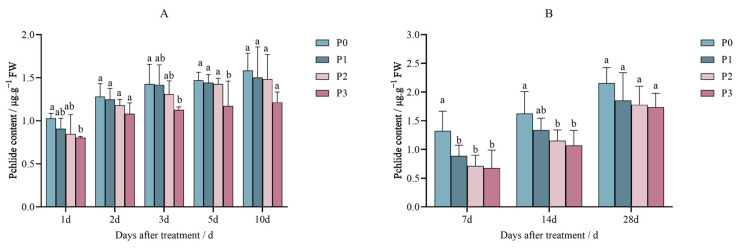
Effect of penoxsulam on Pchlide content of foxtail millet (**A**) pot experiment, (**B**) field experiment. Comparison between treatments of different concentrations on the same day, with lowercase letters representing a significant difference (*p* < 0.05). P0–P3 represent four different spraying doses of 0, 15, 30 and 60 g a.i. Ha^−1^,respectively.

Figure 7A shows the 1st day of the pot experiment; after penoxsulam was sprayed on Jingu 21 leaves, the Pchlide content decreased by 21.90% with the P3 dose compared to P0, while the Pchlide content of the P1 and P2 treatments was not significantly different from that in P0 treatment. In a continuation of the pot experiment, on the 2nd day after penoxsulam was sprayed on Jingu 21 leaves, the Pchlide content showed no significant concentration differences between all dosages sprayed. After the 3rd day of the pot experiment of penoxsulam sprayed on Jingu 21 leaves, the Pchlide content of P3 decreased by 21.04% compared with P0. The Pchlide content of P1 and P2 treatments was not significantly different from that in P0 treatment. After 5 days of the pot experiment of the penoxsulam sprayed on Jingu 21 leaves, Pchlide contents showed no effect under the P1 and P2 dosages compared with P0. Compared with P0, the content of Pchlide in the P3 treatment decreased by 20.32%. At the end of the 10th day of the pot experiment of the penoxsulam sprayed on Jingu 21 leaves, Pchlide content showed no significant differences between P0 and all penoxsulam doses sprayed (Figure 7A).

Figure 7B shows the results of the field experiment after 7 days of penoxsulam sprayed on Jingu 21 leaves; the Pchlide content decreased by 32.85%, 45.93%, and 48.91%, respectively, compared with P0. Furthermore, on the 14th day after penoxsulam was sprayed on Jingu 21 leaves, the Pchlide content decreased by 29.18% and 34.30% under the P2 and P3 sprayed dosages, respectively, compared to P0. The difference in the Pchlide content between P1 and P0 was not significant. Finally, after 28 days of the field experiment of penoxsulam sprayed on Jingu 21 leaves, the Pchlide content showed no significant differences between P0 and the different dosages sprayed (Figure 7B).

### 2.2. Effects of Penoxsulam on Chlorophyll Synthase in Foxtail Millet Leaves

The MgCh activity in Jingu leaves 21 decreased after being sprayed with penoxsulam (P0, P1, P2, and P3), from 27.185 U.g^−1^ to 14.413 U.g^−1^ as the dosage increased.

**Figure 8:**
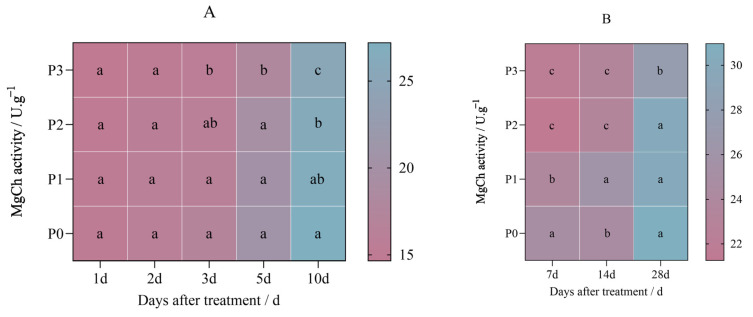
Effect of penoxsulam on MgCh activity of foxtail millet. (**A**) Pot plant experiment; (**B**) field experiment. Comparison between treatments of different concentrations on the same day, with lowercase letters representing a significant difference (*p* < 0.05). P0–P3 represent four different spraying doses of 0, 15, 30 and 60 g a.i. Ha^−1^ respectively.

Figure 8A shows the results on the first day of the pot experiment when the Jingu leaves 21 were sprayed with different penoxsulam dosages; the MgCh activity between P0, P1, P2, and P3 showed no penoxsulam effect. In the pot experiment, after the 2nd day of penoxsulam sprayed on Jingu leaves 21, MgCh activity decreased by 4.91% in the P3 treatment compared with P0. The activity of MgCh under the P1 and P2 treatments was not significant compared with P0. After 3 days of the pot experiment, when the Jingu leaves 21 were sprayed with different penoxsulam dosages, the MgCh activity decreased by 4.60%, 4.77% and 7.98%, respectively, compared with P0. After the 5th day of the pot experiment, when the Jingu leaves 21 were sprayed with different penoxsulam dosages, the MgCh activity decreased by 12.06% under the P3 treatment compared with P0. The activity of MgCh under the P1 and P2 treatments was not significant compared with P0. On the 10th day of the pot experiment of the Jingu leaves 21 sprayed with different penoxsulam dosages, the MgCh activity decreased by 3.42% and 7.01% under the P2 and P3 dosages, respectively, compared with P0. The activity of MgCh under the P1 treatment was not significant compared with P0 (Figure 8A).

Figure 8B shows the results of the field experiment when the Jingu leaves 21 were sprayed with penoxsulam dosages. In the field experiment, after 7 days of Jingu leaves 21 sprayed with different penoxsulam dosages, the MgCh activity decreased by 4.91%, 15.46%, and 11.94%, respectively, compared with P0. On the 14th day of the field experiment of penoxsulam sprayed on Jingu 21 leaves, the activity of MgCh decreased by 5.17% under the P1 dosage compared with P0. Compared with P0, the activity of MgCh in the P2 and P3 treatments decreased significantly by 5.95% and 5.44%. Finally, after the 28th day of the field experiment with different penoxsulam dosages sprayed on Jingu 21 leaves, there was no effect in the MgCh activity among the P1, P2, and P0 sprayed dosages. The activity of MgCh under the P3 treatment decreased significantly by 10.72% compared with P0 (Figure 8B).

### 2.3. Effects of Penoxsulam on Chlorophyll Degrading Enzymes in Foxtail Millet

The PAO activity in Jingu 21 increased after the treatment with penoxsulam (P0, P1, P2, and P3), and the increasing trend was gradual with the increase in the spraying dose.

**Figure 9:**
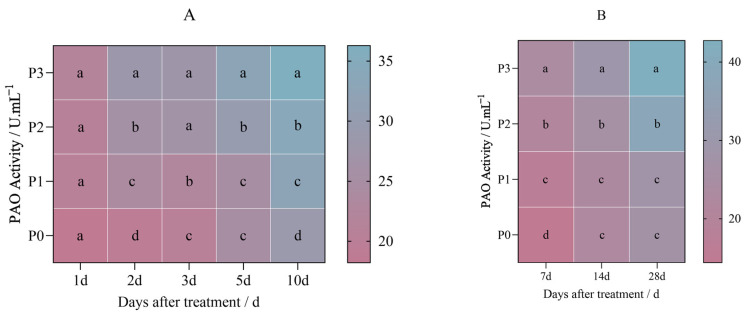
Effect of penoxsulam on PAO activity of foxtail millet. (**A**) Pot plant experiment; (**B**) field experiment. Comparison between treatments of different concentrations on the same day, with lowercase letters representing a significant difference (*p* < 0.05). P0–P3 represent four different spraying doses of 0, 15, 30 and 60 g a.i. Ha^−1^ respectively.

Figure 9A shows the results of the pot experiment after the first day of penoxsulam dosages sprayed on Jingu 21 leaves; there were no significant differences in the PAO activity between P0 and different spraying doses on the 1st day after spraying. The PAO activity increased by 26.97%, 37.45%, and 48.45% compared with that of P0 on the 2nd day after treatment. Similarly, the PAO activity increased by 11.89%, 31.16%, and 34.81% compared with that of P0 at 3 days after treatment. On the 5th day after treatment, the PAO activity showed no significant differences between the P1 and P0 spraying doses, while the PAO activity under the P2 and P3 treatments increased by 20.02% and 32.29%. After 10 days of treatment, the PAO activity increased by 13.14%, 16.85%, and 25.84% compared with that under P0 (Figure 9A).

**Figure 10:**
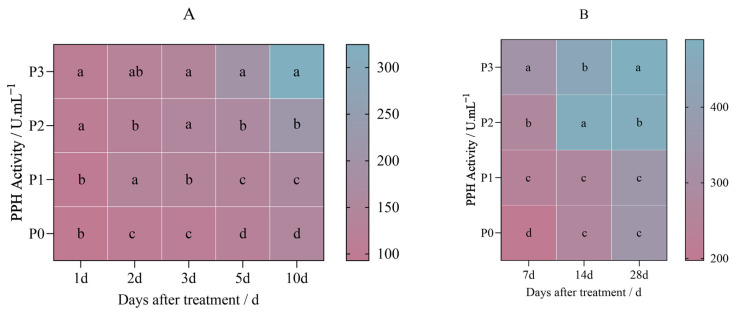
Effect of penoxsulam on PPH activity of foxtail millet. (**A**) Pot plant experiment; (**B**) field experiment. Comparison between treatments of different concentrations on the same day, with lowercase letters representing a significant difference (*p* < 0.05). P0–P3 represent four different spraying doses of 0, 15, 30 and 60 g a.i. Ha^−1^ respectively.

Figure 10B shows the results of the field experiment after 7 days of penoxsulam dosages sprayed on Jingu 21 leaves; the PAO activity increased by 19.02%, 42.04%, and 63.95% compared with that under P0. On the 14th day of the field experiment with penoxsulam dosages sprayed on Jingu 21 leaves, the PAO activity increased by 16.55% and 32.31% under the P2 and P3 dosages compared with P0. There was no significant difference in PAO activity between P1 and P0. However, at 28 days of the field experiment of penoxsulam dosages sprayed on Jingu 21 leaves, there were no significant differences in the PPH activity between P1 and P0 doses. Compared with P0, PPH activity under the P2 and P3 treatments increased significantly by 39.77% and 58.78%, respectively (Figure 9B).

After the penoxsulam (P0, P1, P2, and P3) treatment, the activity of PPH in Jingu 21 increased, and the increasing trend was gradual with an increasing spraying dose.

Figure 10A shows the results of the pot experiment after the first day of penoxsulam dosages sprayed on Jingu 21 leaves; the PPH activity increased by 14.54% and 15.85% under P2 and P3 dosages, respectively, compared with P0. There was no significant difference in PPH activity between P1 and P0. On the 2nd day of the pot experiment of penoxsulam dosages sprayed on Jingu 21 leaves, the PPH activity increased by 26.85%, 19.07%, and 22.04%, respectively, compared with P0. After 3 days of the pot experiment of penoxsulam dosages sprayed on Jingu 21 leaves, the PPH activity increased by 22.89%, 31.52%, and 23.70%, respectively, compared with P0. After 5 days of the pot experiment of penoxsulam dosages sprayed on Jingu 21 leaves, compared with P0, the PPH activity under the P1, P2 and P3 treatments increased by 8.31%, 28.15% and 58.63%, respectively. After 10 days of the pot experiment of penoxsulam dosages sprayed on Jingu 21 leaves, the PPH activity increased by 6.47%, 45.42%, and 120.88%, respectively, compared with P0 (Figure 10A).

Figure 10B shows the results of the field experiment after 7 days of penoxsulam dosages sprayed on Jingu 21 leaves; the PPH activity increased by 17.89%, 36.99%, and 263 64.74% compared with P0. On the 14th day of the field experiment of penoxsulam dosages sprayed on Jingu 21 leaves, the PPH activity increased by 78.29% and 64.86% under the P2 and P3 dosages, respectively, compared with P0. There was no significant difference in PPH activity between P1 and P0. However, at 28 days of the field experiment of penoxsulam dosages sprayed on Jingu 21 leaves, there were no significant differences in the PPH activity between P1 and P0 doses. Compared with P0, PPH activity under the P2 and P3 treatments increased significantly by 35.11% and 39.95%, respectively (Figure 10B).

## 3. Discussion

Chlorophyll absorbs, transmits and converts light energy, and its content is an important index of chloroplast development and photosynthetic capacity [36]. Soares et al. [37] showed that under glyphosate stress, the contents of total chlorophyll and carotenoids in tomato leaves decreased significantly. The contents of Chl a, Chl b, and carotenoids in foxtail millet decreased under low light at the grain-filling stage [38]. Guo et al. [39] reported that the chlorophyll content of foxtail millet decreased significantly under 2-methyl-4-chlorophenoxyacetic acid stress; however, the application did not cause a continuous decrease.

This study showed that after spraying penoxsulam, the content of plant chlorophyll synthesis precursors (such as ALA, protoporphyrin IX, etc.) dropped significantly. The activity of key chlorophyll synthase (MgCh) was inhibited. The activities of chlorophyll degrading enzymes (PAO and PPH) increased significantly. This result reveals that penoxsulam interferes with the steady state of chlorophyll metabolism, which may lead to the decrease of photosynthetic capacity of plants.

Chlorophyll biosynthesis begins with the transformation of glutamic acid into δ -aminolevulinic acid (ALA), which is catalysed by glutamyl -tRNA reductase (GluTR) [20]. The synthesis process of ALA and Mg-ProtoIX is two key rate-limiting steps in the chlorophyll synthesis pathway, and its synthesis rate and efficiency directly affect the synthesis efficiency of chlorophyll a and chlorophyll b [40]. This study found that the content of ALA decreased significantly after penoxsulam treatment, indicating that it may directly or indirectly inhibit the activity of GluTR, thus inhibiting chlorophyll synthesis. Zhao et al. [41] studied chlorophyll synthesis during rice turning green at low temperature, and concluded that chlorophyll biosynthesis was inhibited and chlorophyll accumulation was reduced because of the inhibition of ALA synthesis, which was consistent with the results of this study. Linu et al. [42] reported that higher doses of penoxsulam reduced the chlorophyll (a and b) content in rice. Netherland et al. [43] reported that penoxsulam inhibited chlorophyll a formation in algae.

Further analysis showed that the protoporphyrin IX(ProtoIX) level also decreased significantly. ProtoIX is the common precursor of chlorophyll and heme synthesis, and the decrease in its content may be due to the unbalanced competitive distribution between iron chelatase (FC) and magnesium chelatase (MgCh) [44]. MgCh catalyzes the combination of protoporphyrin and magnesium ions in the chlorophyll biosynthesis pathway to produce Mg-protoporphyrin IX (Figure 1). The inhibition of synthesis will lead to a decrease in chlorophyll content and a change in leaf colour [45]. It is reported that the mutation of related genes led to a significant decline in MgCh activity and chlorophyll synthesis [45,46]. Combined with the experimental data of decreased MgCh activity, it is speculated that penoxsulam may preferentially inhibit MgCh activity, leading to ProtoIX shunting to the heme pathway (Figure 1), thus aggravating the chlorophyll synthesis disorder.

In the process of chlorophyll degradation, Magnesium dechelet alase (PAO) and Chlorophyllase (CLH) are key enzymes. It is generally believed that the higher their activities, the faster the chlorophyll degradation will be [47,48]. PPH is a serine hydrolase, which has the ability to hydrolyse magnesium chlorophyll specifically. If PPH is mutated, the leaves will have a persistent green phenotype because chlorophyll cannot be degraded [49]. PAO plays a key role in the process of chlorophyll degradation, and its enzyme activity, protein abundance and gene expression are positively correlated with the degradation rate of chlorophyll [50] (Figure 2). The deletion of the PAO gene will completely inhibit the decomposition of chlorophyll at the Pheide a level [51]. Ghandchi et al. [52] found that transcription factors, such as ATAF1, in plants can regulate chlorophyll degradation by up-regulating PAO expression under environmental stresses, such as high temperature, ageing and drought. In this study, the activity of the chlorophyll synthase system (MgCh) generally decreased, while the activity of degrading enzymes (PAO, PPH) increased, showing a typical “synthesis inhibition–degradation promotion” double-path interference mode.

The disorder of chlorophyll metabolism directly leads to the damage of the photosynthetic electron transfer chain. Previous studies have observed that the maximum photochemical efficiency *(Fv*/*Fm*) of PSII decreased after spraying penoxsulam [53], which confirmed that the obstacle of chlorophyll synthesis was an important cause of photosynthetic inhibition. This discovery provides a new perspective to explain the weeding effect of penoxsulam: besides the classical ALS inhibition, its secondary damage to photosynthetic organs may accelerate plant death.

There are still the following limitations in this study: firstly, the determination of enzyme activity is based on an in vitro system, which fails to reflect the influence of intracellular localisation and covalent modification. Secondly, it is not clear whether penoxsulam directly acts on the promoter region of chlorophyll metabolism-related genes. Future research can combine ChIP-seq technology to analyse the epigenetic regulation mechanism or use genetic mutants to verify the functional response of key enzymes. In addition, whether the exogenous addition of ALA or antioxidants can reverse the disorder of chlorophyll metabolism is worthy of further discussion.

## 4. Materials and Methods

### 4.1. Materials

The foxtail millet variety used was Jingu 21 (bred by the Economic Crops Research Institute of Shanxi Agricultural University). Similarly, 25 g/L penoxsulam was purchased from Nantong Jinling Agrochemical Co., Ltd., Nantong, China.

### 4.2. Experimental Design

Pot experiment: The experiment was conducted at the Crop Chemical Regulation Laboratory of Shanxi Agricultural University. A completely random design was adopted to select full and uniform Jingu 21 seeds, which were sown in a nutrient bowl with a substrate (5 cm long, 5 cm wide, and 10 cm high) and cultured in an artificial climate room. The greenhouse was illuminated under a 16:8 h (light/dark) cycle, the temperature was 25:18 °C (light/dark), the illuminated intensity was 12,000 lx, and the relative humidity was 70–80%. When foxtail millet was grown to three leaves and one heart, seedling thinning and soil cultivation were performed, excluding five seedlings. When the seedlings grew five leaves and one heart, three doses of penoxsulam were sprayed on the leaves using a 3WP-2000 walking spray tower (nozzle flow rate 390 mL·min^−1^, spray height 300 mm, effective spray width 350 mm, developed by Nanjing Institute of Agricultural Mechanization, Ministry of Agriculture and Rural Affairs, Nanjing, China). The dosage is presented in Table 1. The same amount of clear water (P0) was sprayed as the control. Potted plants are regularly replenished with water by bottom irrigation. The investigation was performed 1, 2, 3, 5, and 10 days after spraying.

Field experiment: The field experiment site was at the Shanxi Agricultural University, Jinzhong City, Shanxi Province. In the 0–20 cm soil layer with a pH of 8.15, the total phosphorus, total potassium, total nitrogen, available potassium, alkali-hydrolyzable nitrogen, available phosphorus, and organic matter contents were 1.134, 22.2, 1.053, 314, 68.5, 18.8, and 25.1 g/kg, respectively. On 23 May 2023, Jingu 21 was planted in a representative plot, and field management events, such as sowing, fertilisation, and watering, were conducted according to local standards. One to two days before sowing, 750 kg/hm^2^ of compound fertiliser was applied as the base fertiliser and soil preparation was carried out. No fertiliser was applied during the growth period of foxtail millet. On 23 June 2023, penoxsulam was sprayed at the five-leaf, one-heart stage of foxtail millet. The dosage is presented in Table 1. The same amount of clear water (P0) was sprayed as a control. Field investigations were performed 7, 14, and 28 days after the spraying treatment.

### 4.3. Analysis of Test Indicators and Methods

In each treatment, five Jingu 21 plants with consistent growth were selected. Two leaves were removed, the middle section of the leaves was cut, the main veins were removed, cut, and mixed evenly, and specific indices were measured, as described in the subsequent sections. Refer to Figure 1 and Figure 2 for the measurement index.

The ALA content was determined according to the approach of Dei [34] and other methods. The sample (0.2 g) was ground in liquid nitrogen and placed in a 10 mL centrifuge tube, to which 4 mL of trichloroacetic acid was added, followed by centrifugation at 7000× *g* for 10 min. Subsequently, 1 mL of the supernatant was transferred into a 2 mL centrifuge tube, followed by the addition of 500 μL of 1 mol/L sodium acetate and 50 μL of acetylacetone, which was heated in a boiling water bath for 10 min, cooled, and underwent 7000× *g* centrifugation for 10 min. Next, 1 mL of the supernatant was transferred into a new 2 mL centrifuge tube, followed by the addition of 1 mL of Ehrlich-Hg solution (composition: 42 mL of glacial acetic acid + 8 mL of 70% perchloric acid + 1 g of dimethylaminobenzaldehyde) for colour development, dark reaction for 15 min, and optical density (OD) measurement at 553 nm.

We adopted the method of Bogorad [54] to determine the PBG content. We weighed 0.2 g of the sample in liquid nitrogen, placed it in a 10-mL centrifuge tube, added 5 mL of phosphate buffer (0.6 mol L^−1^ Tris, 0.1 mol L^−1^ EDTA, pH 8.2), centrifuged at 12,000× *g* for 10 min, transferred 1 mL of the supernatant into a 2 mL centrifuge tube, added 1 mL of Ehrlich-Hg solution, developed the solution in the dark for 15 min, and measured the OD at 553 nm with a microplate reader. The relative PBG content was expressed as absorbance per unit mass.

Rebeiz’s [55] approach was used to determine the ProtoIX content. The sample (0.2 g) was weighed, ground in liquid nitrogen, placed in a 10-mL centrifuge tube and centrifuged at 7000× *g* for 10 min. The ODs were measured at 575, 590, and 628 nm. ProtoIX content = 0.18016OD_575_ − 0.04036OD_628_ − 0.04515OD_590_.

Mg-ProtoIX content was determined using the method of Rebeiz [55]. The sample (0.2 g) was weighed, ground in liquid nitrogen, placed in a 10 mL centrifuge tube and centrifuged at 7000× *g* for 10 min. The ODs were determined at 575, 590, and 628 nm. Mg-ProtoIX content = 0.06077OD_590_ − 0.01937OD_575_ − 0.003423OD_628_.

The Pchlide content was determined according to the method of Rebeiz [55]. The ODs of Pchlide were measured at 575, 590, and 628 nm by weighing 0.2 g of the sample and grinding it in liquid nitrogen. Subsequently, the solution was placed in a 10 mL centrifuge tube, to which 5 mL of 80% basic acetone was added followed by centrifuging at 7000× *g* for 10 min and OD measurements at 575, 590, and 628 nm. Pchlide content = 0.03563OD_628_ + 0.007225OD_590_ − 0.02955OD_575_.

According to the instructions in the plant MgCh, PAO, and PPH determination kit, the enzymatic activities of MgCh, PAO, and PPH were determined as follows:

1. To prepare crude enzyme solution, 0.1 g of leaves were added with precooled 50 mM phosphate-buffered saline (pH 7.4), ground in an ice bath to homogenate and transferred into a 1.5 mL centrifuge tube to spin at 4 °C and 3000× *g* for 20 min. The obtained supernatant was the crude enzyme solution.
2. Adding samples: Standard, sample, and control holes were established, and 50 μL of standard samples with different concentration gradients were added to the standard holes. Crude enzyme solution (10 μL) was added to the sample hole, followed by the addition of 40 μL of sample diluent. No reagent was added to the control hole. The samples were added directly to the bottom of the enzyme-labelled plate, shaken, and mixed evenly.
3. Enzyme incubation: An enzyme-labelled reagent (100 μL) was added to each well except the control well, followed by incubation at 37 °C for 60 min.
4. Washing: The concentrated washing liquid was diluted 20 times into a one-time working liquid, the sealing film was removed and the liquid was discarded. Spin drying was performed, followed by the addition of washing liquid to wash for 30 s. This operation was repeated 5 times.
5. Colour development: A and B colour development solutions (50 μL) were added in turn, shaken and mixed evenly and allowed to react at 37 °C for 15 min.
6. Determination: Termination solution (50 μL) was added, and after the reaction was terminated (blue changed to yellow), the absorbance at 450 nm was determined by setting zero in the control hole.

### 4.4. Data Processing

All experiments were conducted in a completely random design and repeated thrice. Similarly, pot experiments were performed thrice. Data were processed using Microsoft Excel 2021 (Microsoft, Redmond, WA, USA) and IBM SPSS Statistics 27 software (SPSS Inc., Chicago, IL, USA). Duncan’s new multiple-range method was used for the analysis of variance and multiple comparisons (*p* < 0.05). Statistical analyses were performed using GraphPad Prism 9 (GraphPad Software, LLC, San Diego, CA, USA) and Origin 2021 (OriginLab, Northampton, MA, USA).

## 5. Conclusions

**Figure 11:**
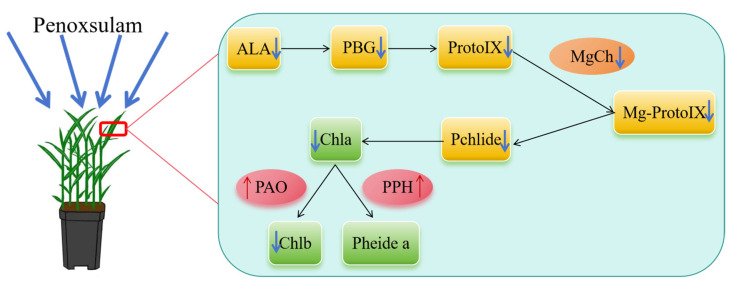
General schematic diagram of the effect of spraying penoxsulam Jingu 21 seedling on chlorophyll biosynthesis and metabolism. An upward red arrow indicates an increasing effect, and a downward blue arrow indicates a decreasing effect.

This study demonstrates that penoxsulam significantly inhibits chlorophyll synthesis and accelerates its degradation in foxtail millet through dual regulation of the chlorophyll metabolism pathway. After the spraying treatment, the content of chlorophyll synthesis precursor material decreased, the activity of MgCh decreased, and the activities of PAO, PPH increased (Figure 11). The results reveal the core mechanism of the drug damage in foxtail millet, which disturbs the photosynthetic system function by breaking the dynamic balance of chlorophyll synthesis and degradation. The experiment confirmed the safety of applying penoxsulam (15 g a.i. Ha^−1^) to foxtail millet, indicating the potential for dose optimisation without damaging plant health. This finding provides an important theoretical basis for the scientific application of herbicides, the breeding of resistant varieties and the prevention and control. It suggests that the regulation network of chlorophyll metabolism gene expression should be further explored.

## Figures and Tables

**Table 1:** Herbicide dosage.

Variety	Treatments	Application Rate/g a.i. Ha^−1^
Jingu 21	P0	0
	P1	15
	P2	30
	P3	60

## Data Availability

The data that support this study are available upon reasonable request from the corresponding authors.

## References

[1] Aggarwal P.R., Pramitha L., Choudhary P., Singh R.K., Shukla P., Prasad M., Muthamilarasan M. (2022). Multi-omics intervention in Setaria to dissect climate-resilient traits: Progress and prospects. Front. Plant Sci..

[2] Muthamilarasan M., Prasad M. (2015). Advances in Setaria genomics for genetic improvement of cereals and bioenergy grasses. Theor. Appl. Genet..

[3] Defelice M. (2002). Green foxtail, *Setaria viridis* (L.) P. Beauv. Weed. Technol..

[4] Pan J., Li Z., Wang Q., Garrell A.K., Liu M., Guan Y., Zhou W., Liu W. (2018). Comparative proteomic investigation of drought responses in foxtail millet. BMC Plant Biol..

[5] Ajithkumar I., Panneerselvam R. (2014). ROS scavenging system, osmotic maintenance, pigment and growth status of *Panicum sumatrense* roth. Under drought stress. Cell Biochem. Biophys..

[6] Zhang G.Y., Liu X., Quan Z.W., Cheng S.F., Xu X., Pan S.K., Xie M., Zeng P., Yue Z., Wang W.L. (2012). Genome sequence of foxtail millet (*Setaria italica*) provides insights into grass evolution and biofuel potential. Nat. Biotechnol..

[7] Singh V., Masabni J., Baumann P., Isakeit T., Matocha M., Provin T., Liu R., Carson K., Bagavathiannan M. (2019). Activated charcoal reduces pasture herbicide injury in vegetable crops. Crop. Prot..

[8] Zhang J., Weaver S.E., Hamill A.S. (2000). Risks and reliability of using herbicides at below-labeled rates. Weed. Technol..

[9] Xu D., Li C., Chen H., Shao B. (2013). Cellular response of freshwater green algae to perfluorooctanoic acid toxicity. Ecotoxicol. Environ. Saf..

[10] Lin L., Chen F., Wang J., Liao M., Lv X., Wang Z., Li H., Deng Q., Xia H., Liang D. (2018). Effects of living hyperaccumulator plants and their straws on the growth and cadmium accumulation of *Cyphomandra betacea* seedlings. Ecotoxicol. Environ. Saf..

[11] Nasir M.U., Hussain S., Jabbar S., Rashid F., Khalid N., Mehmood A. (2015). A review on the nutritional content, functional pro perties and medicinal potential of dates. Sci. Lett..

[12] Liu C., Shi X., Wu F., Ren M., Gao G., Wu Q. (2020). Genome analyses provide insights into the evolution and adaptation of the eukaryotic Picophytoplankton *Mychonastes homosphaera*. BMC Genom..

[13] Ashraf M., Harris P.J.C. (2013). Photosynthesis under stressful environments: An overview. Photosynthetica.

[14] Field K.J., George R., Fearn B., Quick W.P., Davey M.P. (2013). Best of both worlds: Simultaneous high-light and shade-tolerance adaptations within individual leaves of the living stone *Lithops aucampiae*. PLoS ONE.

[15] Gao Y.P., Fang J.G., Zhang J.H., Ren L.H., Mao Y.Z., Li B., Zhang M.L., Liu D.H., Du M.R. (2011). The impact of the herbicide atrazine on growth and photosynthesis of seagrass, *Zostera marina* (L.), seedlings. Mar. Pollut. Bull..

[16] Teodorović I., Knežević V., Tunić T., Cučak M., Lečić J.N., Leovac A., Tumbas I.I. (2012). *Myriophyllum aquaticum* versus Lemna minor: Sensitivity and recovery potential after exposure to atrazine. Environ. Toxicol. Chem..

[17] Wang Q., Que X., Zheng R., Pang Z., Li C., Xiao B. (2015). Phytotoxicity assessment of atrazine on growth and physiology of three emergent plants. Environ. Sci. Pollut. Res..

[18] Nagata N., Tanaka R., Satoh S., Tanaka A. (2005). Identification of a vinyl reductase gene for chlorophyll synthesis in Arabidopsis thaliana and implications for the evolution of Prochlorococcus species. Plant Cell.

[19] Czarnecki O., Grimm B. (2012). Post-translational control of tetrapyrrole biosynthesis in plants, algae, and cyanobacteria. J. Exp. Bot..

[20] Masuda T., Fujita Y. (2008). Regulation and evolution of chlorophyll metabolism. Photochem. Photobiol. Sci..

[21] Chekunova E.M., Yaronskaya E.B., Yartseva N.V., Averina N.G. (2014). New factors regulating magnesium chelatase in the green alga Chlamydomonas reinhardtii. Russ. J. Plant Physiol..

[22] Tanaka R., Tanaka A. (2007). Tetrapyrrole biosynthesis in higher plants. Annu. Rev. Plant. Biol..

[23] Willows R.D. (2003). Biosynthesis of chlorophylls from protoporphyrin IX. Nat. Prod. Rep..

[24] Masuda T., Fusada N., Oosawa N., Takamatsu K., Yamamoto Y.Y., Ohto M., Nakamura K., Goto K., Shibata D., Shirano Y. (2003). Functional analysis of isoforms of NADPH: Protochlorophyllide oxidoreductase (POR), PORB and PORC, in Arabidopsis thaliana. Plant Cell Physiol..

[25] Terry M.J., Smith A.G. (2013). A model for tetrapyrrole synthesis as the primary mechanism for plastid-to-nucleus signaling during chloroplast biogenesis. Front. Plant. Sci..

[26] Sakuraba Y., Schelbert S., Park S.Y., Han S.H., Lee B.D., Andrès C.B., Kessler F., Hörtensteiner S., Paek N.C. (2012). STAY-GREEN and chlorophyll catabolic enzymes interact at light-harvesting complex II for chlorophyll detoxification during leaf senescence in Arabidopsis. Plant Cell.

[27] Hörtensteiner S. (2006). Chlorophyll degradation during senescence. Annu. Rev. Plant. Biol..

[28] Hauenstein M., Christ B., Das A., Aubry S., Hörtensteiner S. (2016). A role for TIC55 as a hydroxylase of phyllobilins, the products of chlorophyll breakdown during plant senescence. Plant Cell.

[29] Hörtensteiner S. (2013). Update on the biochemistry of chlorophyll breakdown. Plant. Mol. Biol..

[30] Pruzinská A., Tanner G., Anders I., Roca M., Hörtensteiner S. (2003). Chlorophyll breakdown: Pheophorbide a oxygenase is a Rieske-type iron-sulfur protein, encoded by the accelerated cell death 1 gene. Proc. Natl. Acad. Sci. USA.

[31] Schelbert S., Aubry S., Burla B., Agne B., Kessler F., Krupinska K., Hörtensteiner S. (2009). Pheophytin pheophorbide hydrolase (pheophytinase) is involved in chlorophyll breakdown during leaf senescence in Arabidopsis. Plant Cell.

[32] Jiang H., Li M., Liang N., Yan H., Wei Y., Xu X., Liu J., Xu Z., Chen F., Wu G. (2007). Molecular cloning and function analysis of the stay green gene in rice: Molecular cloning and function analysis of the rice stay green gene. Plant J..

[33] Li J., Hu L., Zhang L., Pan X., Hu X. (2015). Exogenous spermidine is enhancing tomato tolerance to salinity-alkalinity stress by regulating chloroplast antioxidant system and chlorophyll metabolism. BMC Plant Biol..

[34] Dei M. (1985). Benzyladenine-induced stimulation of 5-aminolevulinic acid accumulation under various light intensities in levulinic acid-treated cotyledons of etiolated cucumber. Physiol. Plant.

[35] Shimoda Y., Ito H., Tanaka A. (2016). Arabidopsis STAY-GREEN, Mendel’s Green Cotyledon Gene, Encodes Magnesium-Dechelatase. Plant Cell.

[36] Woodson J.D. (2019). Chloroplast stress signals: Regulation of cellular degradation and chloroplast turnover. Curr. Opin. Plant Biol..

[37] Soares C., Pereira R., Martins M., Tamagnini P., Serôdio J., Moutinho-Pereira J., Cunha A., Fidalgo F. (2020). Glyphosate-dependent effects on photosynthesis of *Solanum lycopersicum* L.—An ecophysiological, ultrastructural and molecular approach. J. Hazard. Mater..

[38] Yuan X.Y., Zhang L.G., Huang L., Qi X., Wen Y.Y., Dong S.Q., Song X.E., Wang H.F., Guo P.Y. (2017). Photosynthetic and physiological responses of foxtail millet (*Setaria italica* L.) to low-light stress during grain-filling stage. Photosynthetica.

[39] Guo M.J., Bai Y.Q., Gao P., Shen J., Dong S.Q., Yuan X.Y., Guo P.Y. (2020). Effect of MCPA on leaf senescence and endogenous hormones content in leaves of foxtail millet seedlings. Scientia. Agric. Sin..

[40] Cornah J.E., Terry M.J., Smith A.G. (2003). Green or red: What stops the traffic in the tetrapyrrole pathway?. Trends Plant Sci..

[41] Zhao Y., Han Q., Ding C., Huang Y., Liao J., Chen T., Feng S., Zhou L., Zhang Z., Chen Y. (2020). Effect of low temperature on chlorophyll biosynthesis and chloroplast biogenesis of rice seedlings during greening. Int. J. Mol. Sci..

[42] Linu C., Girija T. (2020). Physiological response of rice to herbicide application. Indian. J. Weed. Sci.

[43] Netherland M.D., Lembi C.A., Glomski L.M. (2009). Potential for selective activity of the ALS Inhibitors penoxsulam, bispyribac-sodium, and imazamox on algae responsible for harmful blooms. J. Aquat. Plant Manag..

[44] Tanaka R., Kobayashi K., Masuda T. (2011). Tetrayrrole metabolism in *Arabidopsis thaliana*. Arab. Book..

[45] Zhang H., Li J., Yoo J.H., Yoo S.C., Cho S.H., Koh H.J., Seo H.S., Paek N.C. (2006). Rice Chlorina-1 and Chlorina-9 encode ChlD and ChlI subunits of Mg-chelatase, a key enzyme for chlorophyll synthesis and chloroplast development. Plant. Mol. Biol..

[46] Goh C.H., Satoh K.J., Kikuchi S.S., Kim S.C., Ko S.M., Kang H.G., Jeon J.S., Kim C.S., Park Y.I. (2010). Mitochondrial activity in illuminated leaves of chlorophyll deficient mutant rice (OsCHLH) seedlings. Plant. Biotechnol. Rep..

[47] Fukura K., Tanaka A., Tanaka R., Ito H. (2021). Enrichment of chlorophyll catabolic enzymes in grana margins and their cooperation in catabolic reactions. J. Plant. Physiol..

[48] Dey D., Dhar D., Fortunato H., Obata D., Tanaka A., Tanaka R., Basu S., Ito H. (2021). Insights into the structure and function of the ratelimiting enzyme of chlorophyll degradation through analysis of a bacterial Mg-dechelatase homolog. Comput. Struct. Biotechnol. J..

[49] Zhang J., Yu G., Wen W., Ma X., Xu B., Huang B. (2016). Functional characterization and hormonal regulation of the PHEOPHYTINASE gene LpPPH controlling leaf senescence in perennial ryegrass. J. Exp. Bot..

[50] Pruzinská A., Tanner G., Aubry S., Anders I., Moser S., Müller T., Ongania K.H., Kräutler B., Youn J.Y., Liljegren S.J. (2005). Chlorophyll breakdown in senescent Arabidopsis leaves. Characterization of chlorophyll catabolites and of chlorophyll catabolic enzymes involved in the degreening reaction. Plant. Physiol..

[51] Roca M., James C., Pruzinská A., Hörtensteiner S., Thomas H., Ougham H. (2004). Analysis of the chlorophyll catabolism pathway in leaves of an introgression senescence mutant of Lolium temulentum. Phytochemistry.

[52] Ghandchi F.P., Caetano-Anolles G., Clough S.J., Ort D.R. (2016). Investigating the control of chlorophyll degradation by genomic correlation mining. PLoS ONE.

[53] Dong S., Chen T., Xi R., Gao S., Li G., Zhou X., Song X., Ma Y., Hu C., Yuan X. (2024). Effects of penoxsulam on photosynthetic characteristics and safety evaluation of foxtail millet. Agronomy.

[54] Bogorad L. (1962). Porphyrin synthesis. Methods Enzymol..

[55] Rebeiz C., Mattheis J., Smith B., Rebeiz C., Dayton D. (1975). Chloroplast biogenesis: Biosynthesis and accumulation of protochlorophyll by isolated etioplasts and developing chloroplasts. Arch. Biochem. Biophys..

